# Using a lactadherin-immobilized silicon surface for capturing and monitoring plasma microvesicles as a foundation for diagnostic device development

**DOI:** 10.1007/s00216-020-02938-5

**Published:** 2020-09-22

**Authors:** Agnieszka Kamińska, Katarzyna Gajos, Olga Woźnicka, Anna Dłubacz, Magdalena E. Marzec, Andrzej Budkowski, Ewa Ł. Stępień

**Affiliations:** 1 Department of Medical Physics, M. Smoluchowski Institute of Physics, Jagiellonian University, 30-348 Kraków, Poland; 2 Department of Molecular and Interfacial Biophysics, M. Smoluchowski Institute of Physics, Jagiellonian University, 30-348 Kraków, Poland; 3 Department of Cell Biology and Imaging, Institute of Zoology and Biomedical Research, Jagiellonian University, 30-387 Kraków, Poland; 4 Department of Advanced Materials Engineering, M. Smoluchowski Institute of Physics, Jagiellonian Univeristy, 30-348 Kraków, Poland; 5 Institute of Physics, Cracow University of Technology, 30-084 Kraków, Poland

**Keywords:** Biosensor, Extracellular vesicles, Lactadherin, Microvesicles, Nanotechnology, Time-of-flight secondary ion mass spectrometry

## Abstract

Microvesicles (MVs) are found in several types of body fluids and are promising disease biomarkers and therapeutic targets. This study aimed to develop a novel biofunctionalized surface for binding plasma microvesicles (PMVs) based on a lab-on-a-chip (LOC) approach. A new lactadherin (LACT)-functionalized surface was prepared and examined for monitoring PMVs. Moreover, two different strategies of LACT immobilization on a silicon surface were applied to compare different LACT orientations. A higher PMV to LACT binding efficiency was observed for LACT bonded to an αvβ3 integrin–functionalized surface compared with that for LACT directly bonded to a glutaraldehyde-modified surface. Effective binding of PMVs and its components for both LACT immobilization strategies was confirmed using spectral ellipsometry and time-of-flight secondary ion mass spectrometry methods. The proposed PMV capturing system can be used as a foundation to design novel point-of-care (POC) diagnostic devices to detect and characterize PMVs in clinical samples.

**Electronic supplementary material:**

The online version of this article (10.1007/s00216-020-02938-5) contains supplementary material, which is available to authorized users.

**Figure:**
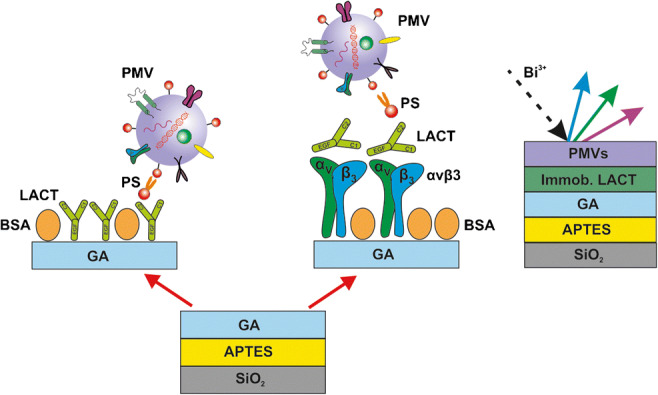
Graphical Abstract

## Background

Microvesicles (MVs), also referred to as ectosomes, are a heterogenous population of extracellular vesicles ranging in diameter from about 100 nm to 1 μm, which arise by outward budding and shedding of the cell membrane [1]. During this process, cell membrane asymmetry is lost, accompanied by the exposure of phosphatidylserine (PS) on the outer membrane layer [2]. Apart from phospholipids, MVs expose different proteins (receptors, glycoproteins) and also carry a variety of other biomolecules such as lipids, nucleic acids, and metabolites, specific to their parental cells. As MVs are present in all body fluids, they have been identified as promising biomarkers for diseases with a diagnostic and prognostic value [3–5]. Especially, use of plasma MVs (PMVs) has been proposed for anti-platelet therapy monitoring in clinical practice [6]. However, the use of appropriate tools for PMV measuring and counting is still a challenge [7, 8]. The main limitation in PMV analysis arises from the problem of obtaining a homogenous fraction of MVs, which is usually affected by different steps in the pre-analytical phase (sample collection, storage) and the diversity of isolation methods (centrifugation, filtration, etc.) [1, 9–12].

To take full advantage of the diagnostic potential of PMVs, fast, reliable, and reproducible methods for their isolation, detection, and characterization are needed. In the last years, lab-on-a-chip (LOC) technologies have attracted much attention, as they have the potential to provide devices based on different approaches (immunoaffinity, size exclusion, or flow-induced) for automated separation and analysis of different populations of extracellular vesicles (EVs) including MVs [13]. In a previous study, we showed that PMVs can be immobilized on a modified silicon substrate functionalized with a specific IgM murine monoclonal antibody against human glycoprotein IIb/IIIa complex (PAC-1) [14]. This approach, followed by different analytical techniques, can be developed to replace antibodies with a glycoprotein lactadherin (LACT), also known as Milk Fat Globule-EGF Factor 8 (MFG-E8), that has an affinity to the components present on the MV surface, e.g. phosphatidylserine (PS) and αvβ3 integrin [15]. LACT is a component of milk fat globules and it contains an epidermal growth factor (EGF)–like domain at the N-terminus and two C-discoidin-type lectin domains similar to the phosphatidylserine (PS)-binding domains of coagulation factors V and VIII [16, 17]. The EGF domain of LACT contains the RGD adhesion motif that is recognized by αvβ3 integrin. In turn, the C2 domain has an affinity to PS [15]. The main advantage of LACT is that the binding process of MVs to this protein is not dependent on the presence of Ca^2+^ ions, which are needed for binding Annexin V [16, 18].

The main objective of our study was to develop a novel biofunctionalized surface for the capture of PMVs and their components, for LOC development. To achieve this goal, a silicon-based surface was functionalized with 3-aminopropyltriethoxysilane (APTES), modified with glutaraldehyde (GA), and finally used for immobilization of human LACT molecules to enable capture of PMVs. Specifically, this work compares two different approaches for LACT immobilization, namely, covalent binding to a GA-modified surface and site-specific binding to αvβ3, previously immobilized on a GA-modified surface. The PMV binding efficiency was examined by spectroscopic ellipsometry (SE) and time-of-flight secondary ion mass spectrometry (ToF-SIMS) methods. ToF-SIMS analysis of the surface molecular composition could also provide information about the PMV phospholipid composition. Moreover, the LACT orientation on the surface for both strategies of immobilization was analyzed by ToF-SIMS supported by principal component analysis (PCA). This developed biosensor could be used as a foundation to design a novel point-of-care (POC) diagnostic device for detecting PMVs in clinical samples.

## Methods

### Isolation of PMVs from human plasma

A whole blood sample from a healthy volunteer was collected into a 10-mL S-Monovette® vacutainer (Sarstedt AG, Nümbrecht, Germany) containing 3.2% trisodium citrate anticoagulant. Sample collection was performed by venipuncture of the antecubital vein with a > 21-gauge needle, by applying a light tourniquet. Within 30 min after collection, the blood sample was centrifuged at 165×*g* (Hermle Z300K, Germany) for 10 min at room temperature (RT) and platelet-rich plasma (PRP) was collected. Then, PRP was centrifuged at 2000×*g* for 10 min to remove the residual platelets and apoptotic bodies. The supernatant was collected and aliquoted into 350 μL, placed into microcentrifuge tubes, and centrifuged at 16,000×*g* (Eppendorf 5424R, Germany) for 1.5 h at 4 °C to obtain a pellet of PMVs. After that, 300 μL of the supernatant was discarded and the remaining volume was pooled. The tube containing the PMV fraction was filled up to 1 mL with phosphate-buffered saline (PBS) composed of 0.01 M phosphate buffer, 0.0027 M potassium chloride, and 0.137 M sodium chloride (cat. no. P4417, Sigma-Aldrich, Darmstadt, Germany), and centrifuged again under the same conditions to obtain washed PMVs. After that, the PMV pellet was suspended in 200 μL of PBS and used for further experiments.

#### Ethical considerations

The study was approved by The Bioethics Committee of the Jagiellonian University which accepted all of the project’s protocols and forms, including an information for volunteers form and a consent form (permission no. 122.6120.78.2016). Only healthy volunteers were involved in this study.

### PMV size distribution and concentration analysis

The size distribution and concentration of isolated PMVs were determined using the nanoparticle tracking analysis (NTA) method on the NanoSight NS300 Malvern system (Malvern Panalytical Ltd., UK) equipped with a 488-nm laser. Before measurement, the PMVs were diluted 2000 times in filtered (0.05 μm) deionized water. The assay was performed at RT (25 °C) and 5 videos of 60 s were recorded for the PMV samples. Data were captured and analyzed using NTA 3.3 software with a detection threshold of 5 and a syringe pump speed of 20 μL/min.

### PMV visualization by transmission electron microscopy

For transmission electron microscopy (TEM) imaging, the PMV pellet was fixed with 2.5% GA (cat. no. G5882, Sigma-Aldrich, St. Louis, USA) in 0.1 M cacodylate buffer (cat. no. C4945, Sigma-Aldrich, St. Louis, USA) for 2 h at RT. Then, samples were postfixed in 1% osmium tetroxide solution (1 h), dehydrated by passing through a graded ethanol series, and embedded in PolyBed 812 at 68 °C. Ultrathin sections were collected on 300-mesh grids or on one slot made from copper; the latter was additionally covered with a formvar film. For cutting, the Leica EM UC7 microtome was used. Then, the sections were contrasted using uranyl acetate and lead citrate. For observation, the JEOL JEM 2100HT electron microscope (Jeol Ltd., Tokyo, Japan) was used at an accelerating voltage of 80 kV.

### Silicon surface modification

Silicon substrates with a native SiO_2_ layer were purchased from Si-Mat (GmbH, Germany). Before modification, the substrates were cleaned by sonication in sequence: toluene (POCh, Gliwice, Poland) and ethanol (POCh, Gliwice, Poland) for 10 min. After that, the substrates were treated with oxygen plasma for 30 s for cleaning and hydrophilization. Then, the substrates were silanized by immersion in a 1% APTES (Sigma-Aldrich, Darmstadt, Germany) solution (v/v) in toluene for 10 min, followed by sonification in sequence in toluene and ethanol, and were finally dried under a N_2_ stream and baked for 20 min at 120 °C. For modification with aldehyde groups enabling covalent protein binding on an amino-silanized silicon surface, the substrates were immersed in a 2.5% (v/v) aqueous GA solution for 20 min. Then, the substrates were washed with distilled water and dried under a N_2_ stream. The effective surface modification with APTES silane and glutaraldehyde is confirmed by measurements of the silane layer thickness (1.2 (± 0.1) nm) and by the increase of water contact angle up to ~ 55°. In addition, the glutaraldehyde activation of APTES layer is revealed by comparison of characteristic ToF-SIMS ion signal intensities presented in Electronic Supplementary Material (ESM) Fig. S1.

### Biomolecule immobilization

**Fig. 1:**
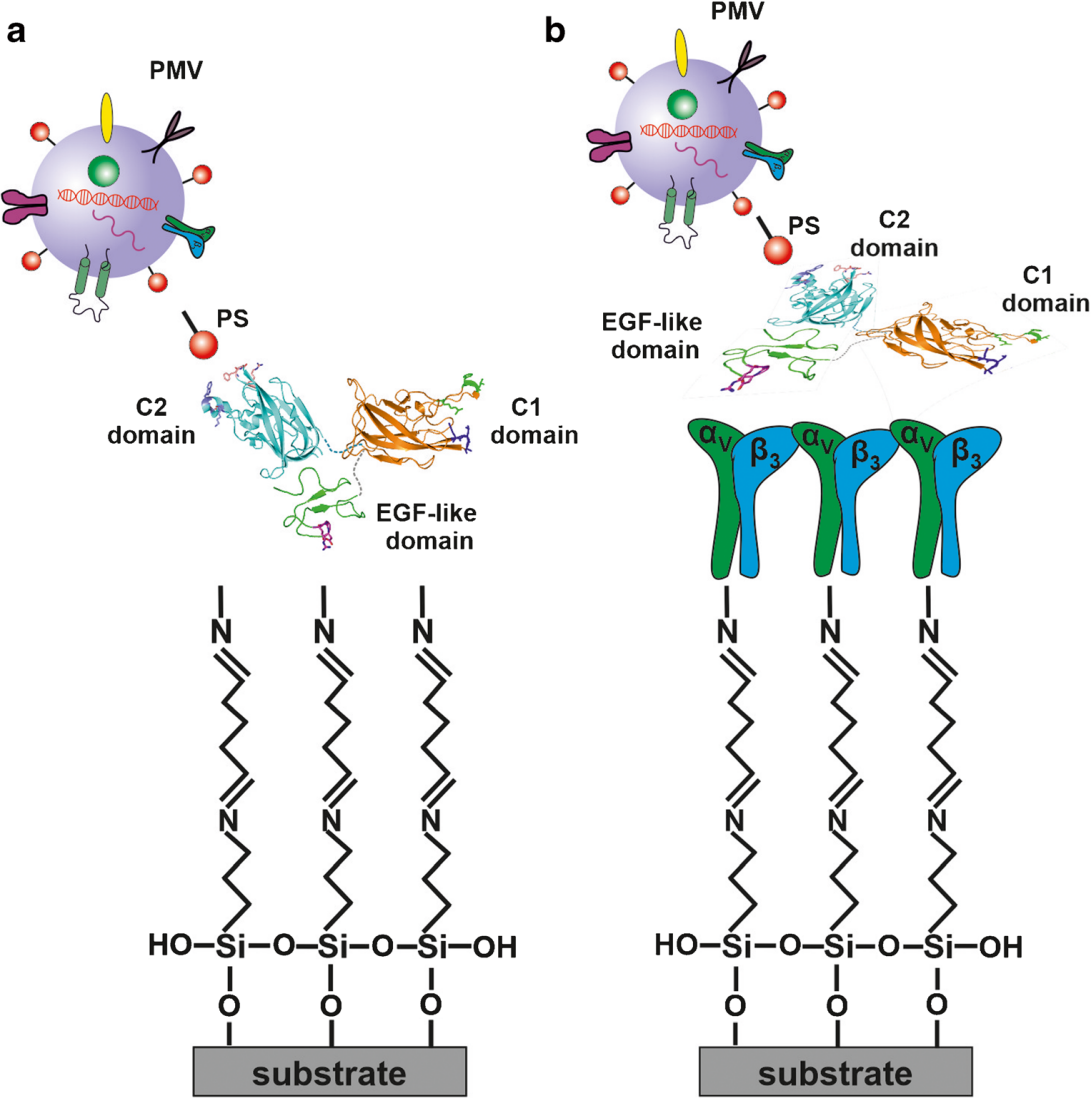
Schema of the two different approaches utilized for functionalizing the silicon surface for plasma microvesicle (PMV) capture: (**a**) covalent binding of lactadherin (LACT) on the surface modified with glutaraldehyde (GA) (side-on orientation of LACT with hidden EGF-like domain) and (**b**) immobilization on the integrin αvβ3–functionalized surface (flat-on orientation of LACT with random domain exposure). The model for EGF-like and C1 domains was generated using homology modeling with Phyre2 [18]. The model for the C2 domain corresponded to the crystal structure of the bovine LACT C2 domain (PDB code: 3BN6). The 3D models of each domain were visualized independently in Pymol 2.3 program [19] and placed arbitrarily

Human recombinant LACT (cat. no. 10853-H08B, Sino Biological) was immobilized by covalent binding on bare GA-modified amino-silanized silicon surface and a human integrin αvβ3 (cat. no. CC1018, Sigma-Aldrich, Darmstadt, Germany)–functionalized surface to compare the different orientations of surface-immobilized LACT (Fig. 1). In order to functionalize the surface with integrin αvβ3, amino-silanized substrates modified with GA were incubated with 50 μg/mL solutions of integrin αvβ3 in PBS, pH 7.4, for 1 h. After that, substrates with covalently bonded αvβ3 were gently washed with PBS and blocked with bovine serum albumin (BSA) (cat. no. BP9706, ACROS Organics, Geel, Belgium) by incubation with a 2 mg/mL BSA solution in PBS for 30 min, followed by washing with PBS. All the prepared functionalized substrates were incubated with a 100 μg/mL LACT solution in PBS for 1 h, for LACT immobilization. After gently washing with PBS, substrates (besides those functionalized with integrin) were blocked with BSA as described above. Additionally, silanized silicon substrates only blocked with BSA protein were prepared as a negative control. Finally, binding of PMVs to surface-immobilized LACT was performed by incubation with PMV solution in PBS for 1 h, followed by washing with PBS. Prior to measurement with surface techniques, all samples were washed with distilled water and dried under a N_2_ stream.

### Determination of the biomolecular surface thickness with SE

To determine the effective ellipsometric thickness of the biomolecular layer on silicon substrates, the Sentech SE800 (Sentech Instruments GmBH) spectroscopic ellipsometer was employed. Measurements were performed over a wavelength range of 320–700 nm and at a fixed angle of incidence equal to 70°. The results were analyzed using SpectraRay 3 software. For estimating the average thickness of the biomolecular layer, the Cauchy dispersion model was assumed and the three-layer model consisting of silicon substrate/mixed SiO_2_ and silane/protein/PMV layers was applied. Fixed refractive index values equal to *n* = 3.87 for Si; *n* = 1.46 for SiO_2_, APTES, and GA [19]; *n* = 1.53 for proteins [20]; and *n* = 1.39 for PMVs [5, 21] were used. A constant thickness of 2.6 nm for the SiO_2_ layer, obtained from fitting measurements performed on the plasma cleaned silicon surface, was also considered to fit the thickness of the layers of silane and biomolecules. Constant values of APTES modified with a GA layer (1.2 (± 0.1) nm) as determined for bare layers were used to fit the thickness of the biomolecular layer.

### Imaging of functionalized surface by atomic force microscopy

Atomic force microscopy (AFM) imaging of the studied surfaces was carried out using an Agilent (Santa Clara, CA, USA) 5500 microscope operating in a non-contact mode. For analysis, AFM tips with constant elasticity of 2 N/m, apex radius < 7 nm, and resonance frequency of about 70 kHz were used. For all samples, the average height distribution in the AFM image and the doubled width in the middle of the maximum radial-averaged function as the AFM height and the size of the feature were adopted.

### ToF-SIMS surface characterization

The TOF.SIMS 5 (ION-TOF GmbH) instrument equipped with Bi_3_^+^ ion clusters (30-keV liquid metal ion gun) was applied for the ToF-SIMS analysis of the surface molecular composition after the subsequent steps of functionalization and PMV binding. An ion dose density of about 10^12^ ion/cm^2^ and a current of about 0.5 pA were applied to all measurements to ensure static mode conditions. A low-energy electron flood gun was used for charge compensation. High mass resolution ToF-SIMS spectra of positive ions were acquired from several non-overlapping 100 μm × 100 μm areas of each sample with an applied resolution of 128 × 128 points. Mass calibration was performed with H^+^, H_2_^+^, CH^+^, C_2_H_2_^+^, and C_4_H_5_^+^ peaks. A minimal mass resolution (m/∆m) > 7500 at C_4_H_5_^+^ was obtained.

#### Principal component analysis

Multivariate principal component analysis (PCA) of the positive ToF-SIMS spectra was performed using the PLS Toolbox (Eigenvector Research, Manson, WA) for MATLAB (MathWorks, Inc., Natick, MA). Before running the PCA, the intensities of selected peaks from each spectrum were normalized to the sum of the selected peaks and mean-centered.

## Results

### PMV size distribution and morphology

**Fig. 2:**
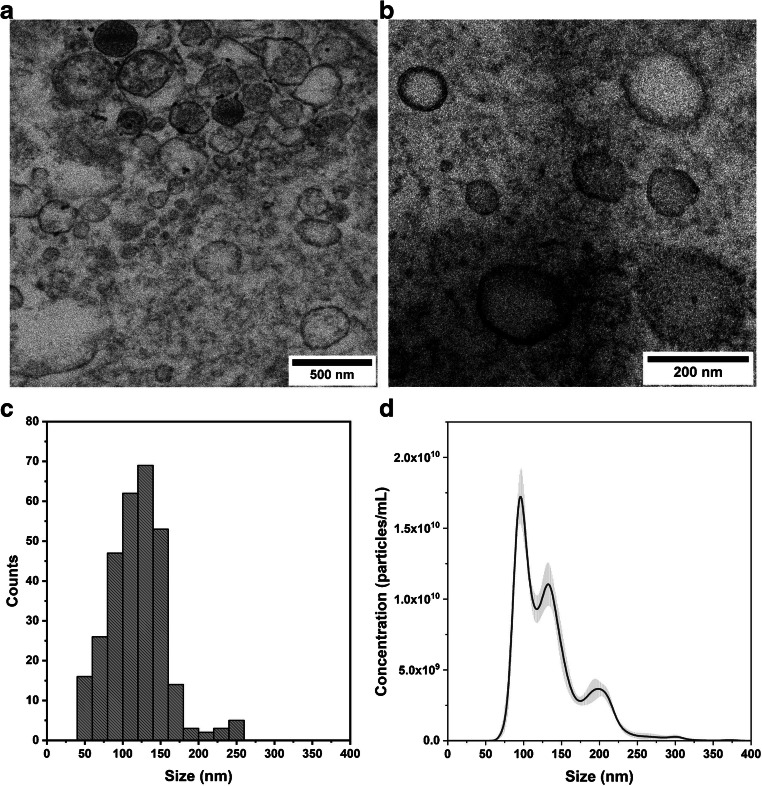
Representative transmission electron microscopy (TEM) images of plasma microvesicles (PMVs) (a, b). The size distribution histogram of PMVs determined fromfive TEM images—measurement of PMV diameter was done in ImageJ software (c). PMV size distribution obtained by the NTA method (d)

The morphology and size distribution of isolated EVs including PMVs were determined by TEM (Fig. 2a–c) and NTA (Fig. 2d), respectively. Fine membrane structures of PMVs contrasted with osmium salt were visible. The obtained TEM images confirmed the preservation of the PMV integrity after the isolation procedure. The mean diameter of PMVs obtained from TEM images was 120 ± 37 nm. The NTA method showed that the isolated population of PMVs had a heterogenous size distribution with an average mean size of 134 ± 45 nm that is in line with the results obtained by Menck et al. [22]. The final concentration of the analyzed sample was equal to 1.08E12 ± 2.38E10 particles/mL. In the separated PMV sample, we observed structures smaller than 100 nm, which may indicate that the sample was slightly contaminated by exosomes.

### Characterization of functionalized surface by atomic force microscopy

**Fig. 3:**
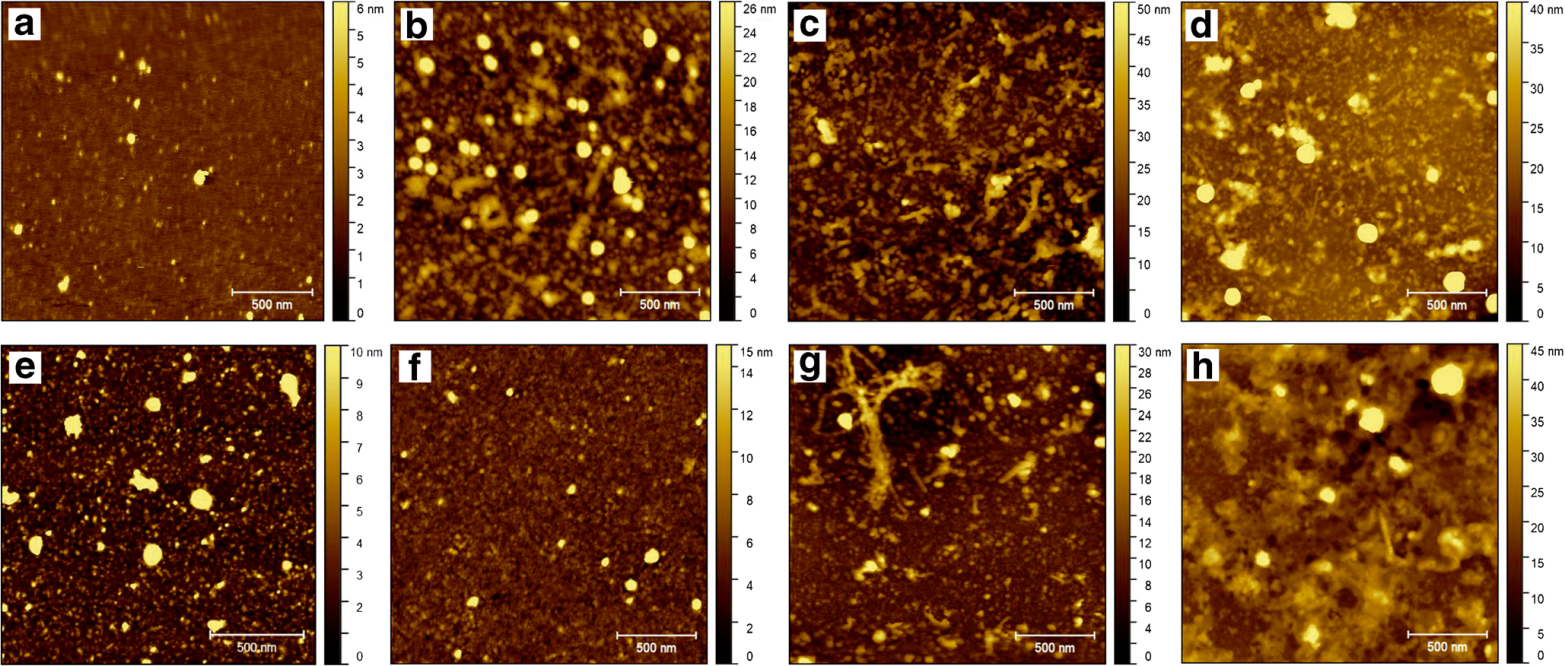
Representative atomic force microscopy topography images of GA-modified surface (**a**) functionalized with LACT (**b**), LACT+BSA (**c**), LACT+BSA+PMVs (**d**), integrin (**e**), integrin+BSA (**f**), integrin+BSA+LACT (**g**), and integrin+BSA+LACT+PMVs (**h**). Colorimetric scale indicates the maximum height detected in each image; scan size is 2 μm × 2 μm

**Fig. 4:**
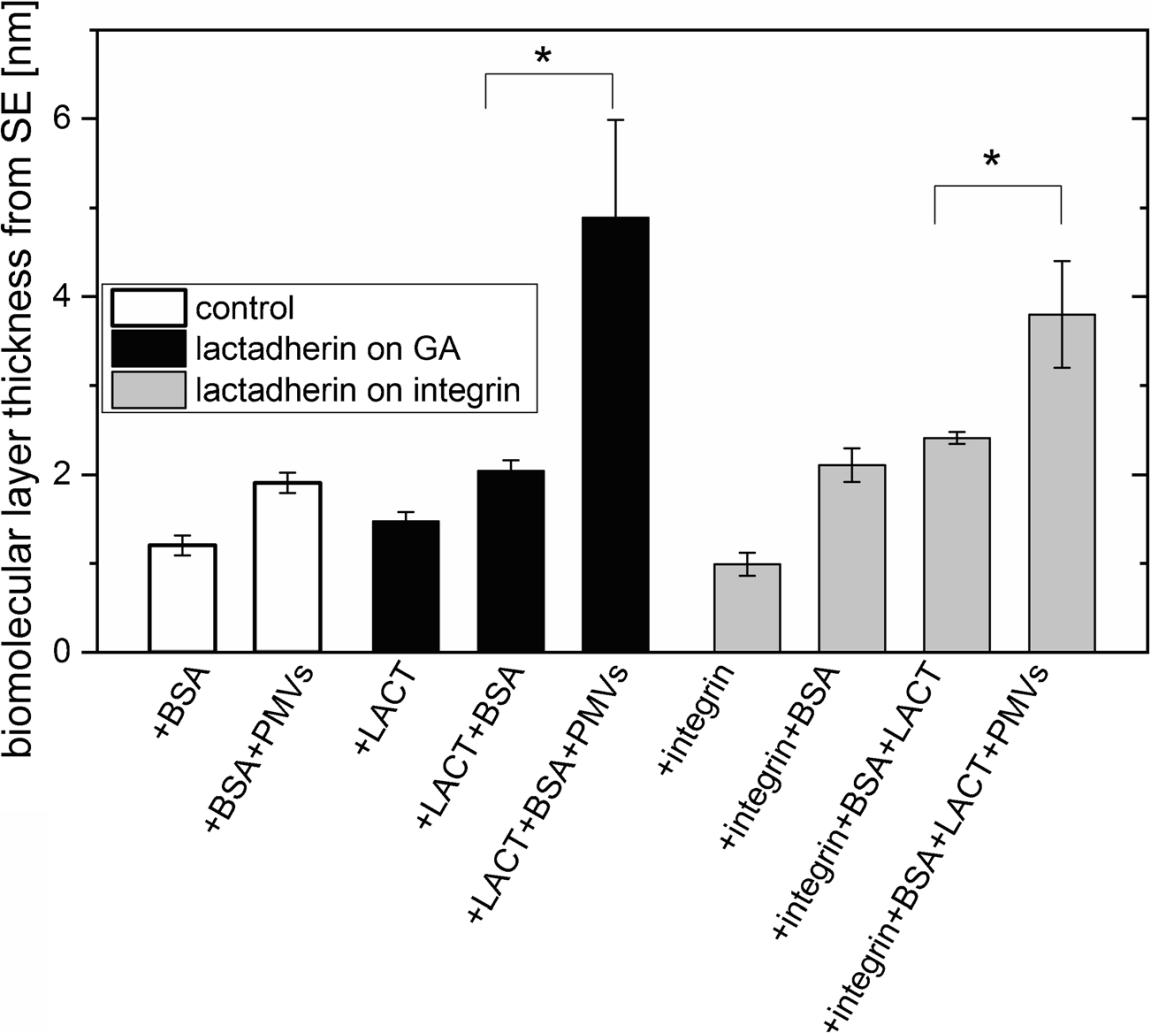
Thickness of biomolecular layers formed on the silicon substrate after lactadherin (LACT) immobilization on bare GA-modified (black columns) and αvβ3 integrin–functionalized surfaces (gray columns) following the protocol of blocking with bovine serum albumin (BSA) and the binding of plasma microvesicles (PMVs). Additionally, the binding of PMVs to the control sample blocked with BSA was tested (white columns). Thickness was determined with SE; error bars are standard deviations from 10 measurements of the same sample. For both the LACT immobilization strategies, the difference in the thickness of biomolecular layers before and after PMV binding was detected (significant differences in the thicknesses were determined by using the ANOVA test, *p* < 0.05 (indicated by the asterisk symbol “*”))

The topography of the functionalized silicon surfaces was imaged using the AFM method. Representative AFM images obtained for selected functionalization steps are presented in Fig. 3. We observed higher density of LACT molecules on the surface for LACT directly bonded to GA-modified surface in comparison with LACT bonded to an αvβ3 integrin–functionalized surface (Fig. 3b and g, respectively). These observations are in line with results described in the “Evaluation of biomolecular layer thickness using SE” section and presented in Fig. 4. Topographical images demonstrated aggregates of proteins (Fig. 3b, c, e–g). AFM imaging of LACT-functionalized surfaces revealed the presence of bonded PMVs on the surface for both LACT immobilization strategies (Fig. 3d, h).

### PMV binding to the LACT-functionalized silicon surface

The molecular weight of human LACT is approximately 46 kDa, and this Y-shaped glycoprotein consists of three domains: C1 and C2 discoidin-type lectin domains and the small EGF-like domain binding an integrin αvβ3 [16, 23, 24]. Therefore, different LACT orientations adopted on the surface could alter the availability of PS and αvβ3 binding sites. For this reason, we applied and compared two approaches for LACT immobilization on the silicon surface. The first approach involves the immobilization of LACT on GA-modified silicon substrates via covalent binding, combined with blocking the free surface sites with BSA. In turn, site-direct immobilization via binding to an αvβ3-functionalized surface was performed in the second method (Fig. 1). Surface functionalization with the αvβ3 integrin was implemented through binding of the integrin to GA-modified silicon substrates and subsequent blocking with BSA. As a result, two sample series of LACT-functionalized surfaces were obtained and examined for PMV capturing capacity.

### Evaluation of biomolecular layer thickness using SE

SE was applied to evaluate the biomolecular coverage of silicon surfaces after subsequent steps of functionalization and PMV capture. The thickness of the biomolecular layer determined for two series of LACT-functionalized substrates and BSA control samples is presented in Fig. 4. LACT immobilization on the GA-modified surface, enabling covalent protein binding, results in high coverage with an average layer thickness of 1.5 (± 0.1) nm corresponding to a surface density equal to 2.0 (± 0.1) mg/m^2^ (estimated using protein mass density 1.37 g/cm^3^ as the scaling factor [25]). In turn, LACT binding to the αvβ3-functionalized surface provides a significantly lower amount of immobilized LACT, estimated at 0.4 (± 0.2) mg/m^2^. The effective αvβ3 integrin surface functionalization in this approach is confirmed by the determined integrin surface density equal to 1.4 (± 0.2) mg/m^2^. In addition, the proper blocking of free surface sites with the BSA solution is manifested by a surface protein coverage of about 2.7–2.9 mg/m^2^ for both surface functionalization strategies, corresponding to nearly a complete protein monolayer [26]. Finally, binding of PMVs and their components to both LACT-functionalized surfaces and the control surface covered with BSA was examined by analyzing the increase in the biomolecular layer thickness upon incubation in the PMV solution. As shown in Fig. 4, effective PMV capturing was observed with both LACT immobilization strategies, involving direct binding to GA and binding to integrin αvβ3, resulting in an increase in layer thickness of about 2.8 and 1.4 nm, respectively. These results indicate the high efficacy of PMV interaction with the LACT-immobilized surface via PS binding by the C2 domain, as concluded for both immobilization schemes. In addition, the PMV to LACT binding efficiency was estimated simply as the ratio of PMV layer thickness to LACT layer thickness, and was significantly higher for LACT bonded to the αvβ3-functionalized surface, 4.6 (± 2.8) than for the surface modified only with GA 1.8 (± 0.8). This difference could be caused by a more pronounced steric hindrance in case of covalently bonded LACT due to a higher surface density and by different access to the C2 domain due to a possible different orientation of LACT on the surface. In addition, incubation of the control sample with PMVs causes only a slight increase in layer thickness. Further analysis by ToF-SIMS excluded the observable PMV presence on this sample and the non-specific PMV adsorption to the protein layer.

### Lipid and amino acid composition analysis of LACT-functionalized surfaces by ToF-SIMS

**Fig. 5:**
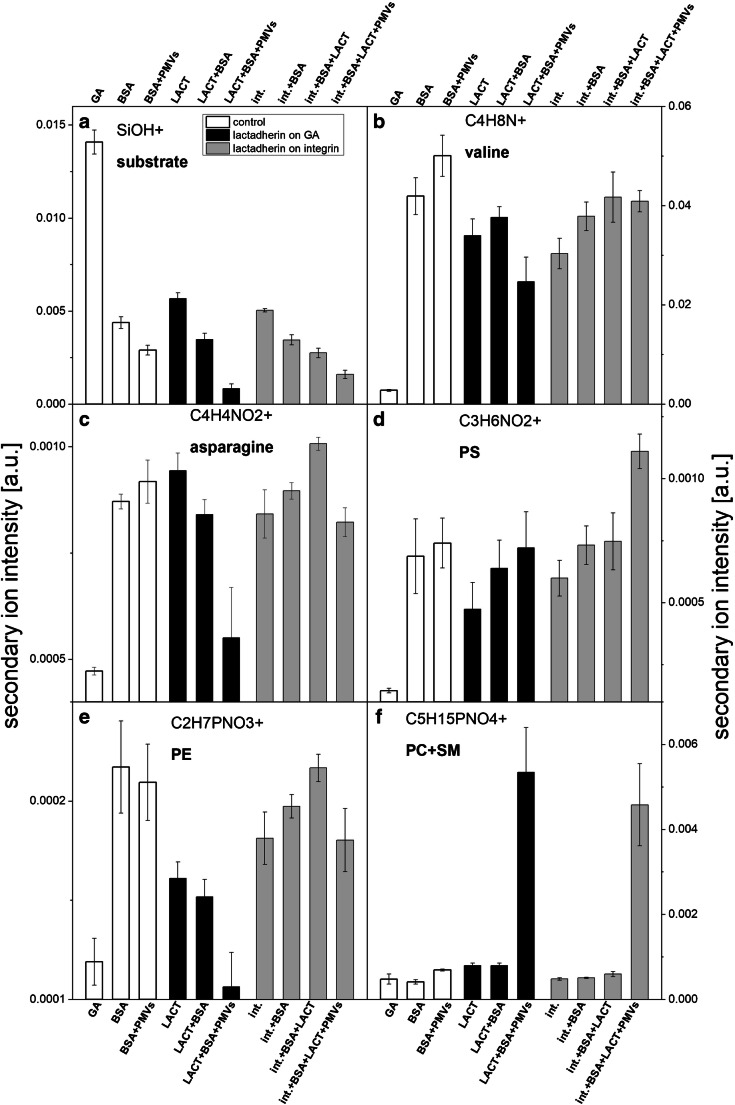
Time-of-flight secondary ion mass spectrometry (ToF-SIMS) analysis of the biomolecular layer composition formed on the silicon substrate after lactadherin (LACT) functionalization on bare GA-modified (black columns) and αvβ3 integrin–functionalized surfaces (gray columns) following the steps of blocking with bovine serum albumin (BSA) and the binding of plasma microvesicles (PMVs). Additionally, the binding of PMVs to the control sample blocked with BSA was tested (white columns). Normalized intensities of secondary ion characteristic for (**a**) substrate (SiOH+) and amino acids abundant in (**b**) all proteins (C4H8N+ from valine) and (**c**) LACT (C4H4NO2+ from asparagine) confirm the effectiveness of subsequent surface functionalization steps. While, normalized intensities of ion fragments derived from different phospholipids: (**d**) phosphatidylserine (PS) (C3H6NO2+), (**e**) phosphatidylethanolamine (PE) (C2H7PNO3+), and (**f**) phosphatidylcholine (PC) and sphingomyelin (SM) (C5H15PNO4+); revealed PMV binding and enabled examination of their lipid composition. Additionally, the binding of PMVs to the control sample blocked with BSA was tested (white columns). Error bars are standard deviations from 10 ToF-SIMS measurements of the same sample

In our study, ToF-SIMS was employed to examine the composition of the biomolecular layers formed on GA-modified silicon substrates after LACT immobilization and the following steps of blocking and PMV binding. This technique combines surface sensitivity with great chemical selectivity allowing for discrimination between different molecules immobilized on the surface, e.g. different proteins [27, 28] and lipids [29–31], based on the differences in their chemical structure, and for comparison of surface molecular composition [32]. Therefore, ToF-SIMS is an excellent method for examination of surface functionalization involving multistep and multimolecular protocols of biosensor surface functionalization [33, 34]. A comparison of the intensities of secondary ion fragments chosen as characteristics for the substrate, all proteins, LACT, and particular lipids is presented in Fig. 5. The ion fragments of amino acid characteristic for all proteins (LACT, BSA, αvβ3) were assigned based on the amino acid composition of particular proteins listed in ESM Table S1. The following signals originating from membrane phospholipids and proteins were identified: C4H8N+ (*m*/*z* = 70) from valine present in all proteins, C4H4NO2+ (*m*/*z* = 98) from asparagine with a uniquely high abundance in LACT, C3H6NO2+ (*m*/*z* 88) from the amino acid serine present in phosphatidylserine (PS), C2H7PNO3+ (*m*/*z* 124) from phosphatidylethanolamine (PE), C5H15PNO4+ (*m*/*z* 184) from phosphocholine present in phosphatidylcholine (PC) and sphingomyelin (SM).

First, reduction of the substrate signal (SiOH+, Fig. 5a) confirms the growth of the biomolecular layer on the substrate upon successive surface functionalization steps and PMV binding. The additional intensity of the C4H8N+ ion signal from valine reflects the successive formation of protein layers including steps such as surface coverage with BSA, LACT immobilization on the GA surface, surface functionalization with αvβ3 integrin, blocking the free surface sites with BSA, and LACT binding to αvβ3 integrin–functionalized surfaces (Fig. 5b). Effective surface functionalization with LACT on both the bare GA-modified surface and αvβ3 integrin–functionalized surface was confirmed by a high signal from asparagine, which is characteristic for this protein (Fig. 5c). Finally, the increase in ion signals originating from lipids and a decrease in those originating from the substrate and proteins demonstrate the efficient surface capture of PMVs for both approaches to surface functionalization with LACT. In addition, no change in any of the lipid-derived signals and a slight increase in the protein-derived signal for the control BSA-covered sample incubated with PMVs clearly exclude the non-specific adsorption of PMVs to the protein layer.

The ability of ToF-SIMS to differentiate membrane lipids combined with the novel method of surface immobilization of PMVs and their components provides a unique opportunity to examine the phospholipids characteristic to PMVs [14]. Enrichment in PS, which is commonly observed in PMVs, was confirmed for both types of LACT-functionalized surfaces (Fig. 5d), reflecting the specific bonding of PS to the LACT C2 domain (Fig. 1). Interestingly, the same configurations of LACT on the surface resulted in a decrease in the PE signal (Fig. 5e), showing that the distribution of phospholipids on the PMV outer layer was depleted due to PE internalization in the course of PMV formation and PS exposure. Analysis of the ion fragment intensities originating from phospholipids indicates the presence of PS, a very high abundance of PC and SM (Fig. 5f), which cannot be distinguished by ToF-SIMS, and a negligible content of PE in the PMV membrane. For both LACT-functionalized surfaces, the magnitudes of the above described changes in the surface composition of each particular phospholipid component are different. This can reflect various exposures of phospholipid components induced by different surface densities and orientations of LACT molecules (Fig. 5d–f).

### PCA of biomolecular layer composition

**Fig. 6:**
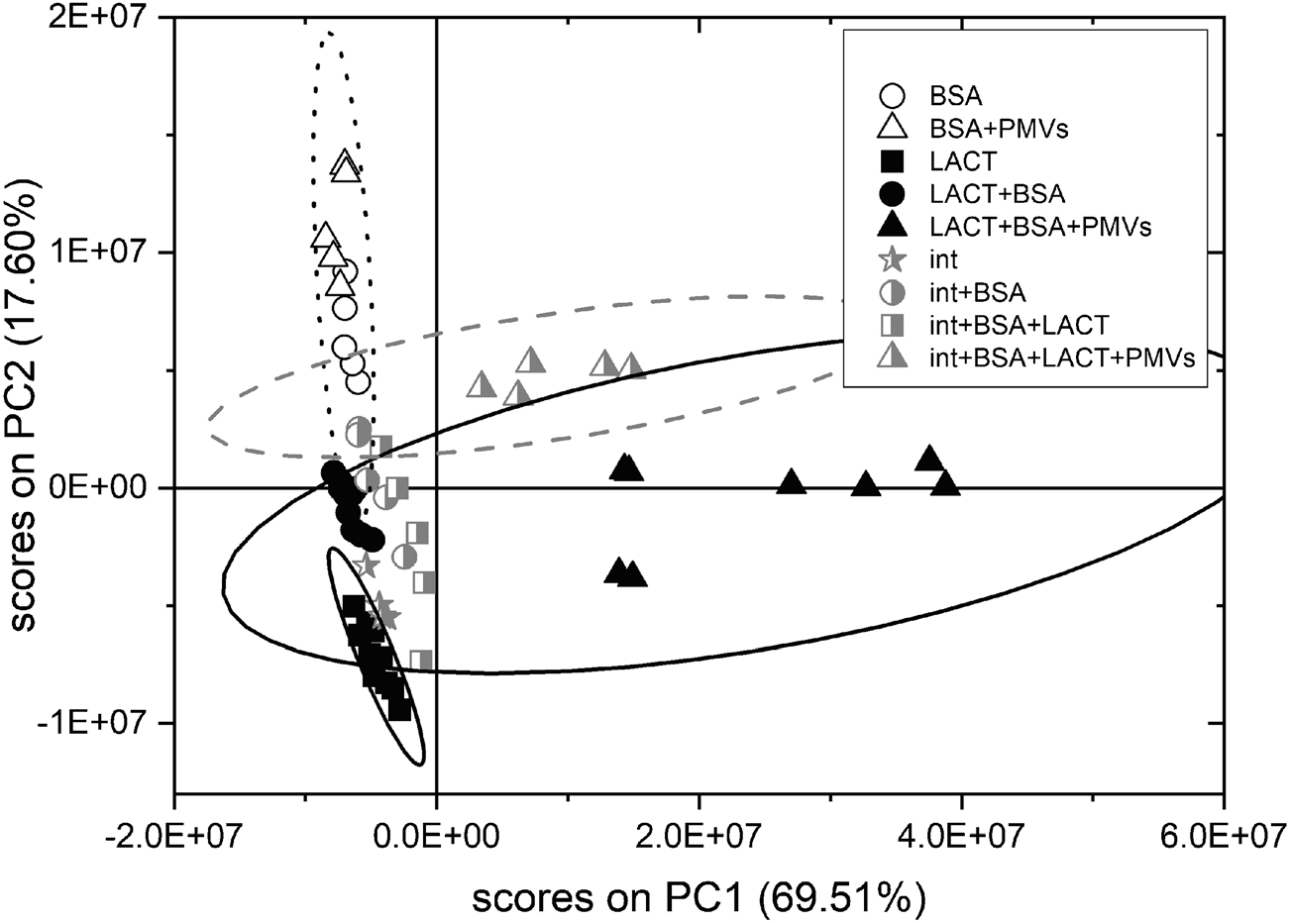
PC1 vs. PC2 scores plot of time-of-flight secondary ion mass spectrometry (ToF-SIMS) spectra recorded from the biomolecular layers formed on the silicon substrate after lactadherin (LACT) functionalization on bare GA-modified (black symbols) and αvβ3 integrin–functionalized surfaces (light gray symbols) following the steps of blocking with bovine serum albumin (BSA) and the binding of plasma microvesicles (PMVs). Additionally, reference samples blocked with BSA were examined (white symbols). PC1 (69.51% of variance) separates protein layers from the abundant layers of PMVs, while PC2 (17.60%) distinguishes protein composition rich in BSA and LACT (see also loading plots). Therefore, the location of data points corresponding to PMVs exposed samples confirms PMVs binding to both lactadherin-functionalized surfaces and exclude a PMV non-specific adsorption (compare the reference BSA-covered surface). The ellipses represent the 95% confidence limit

**Fig. 7:**
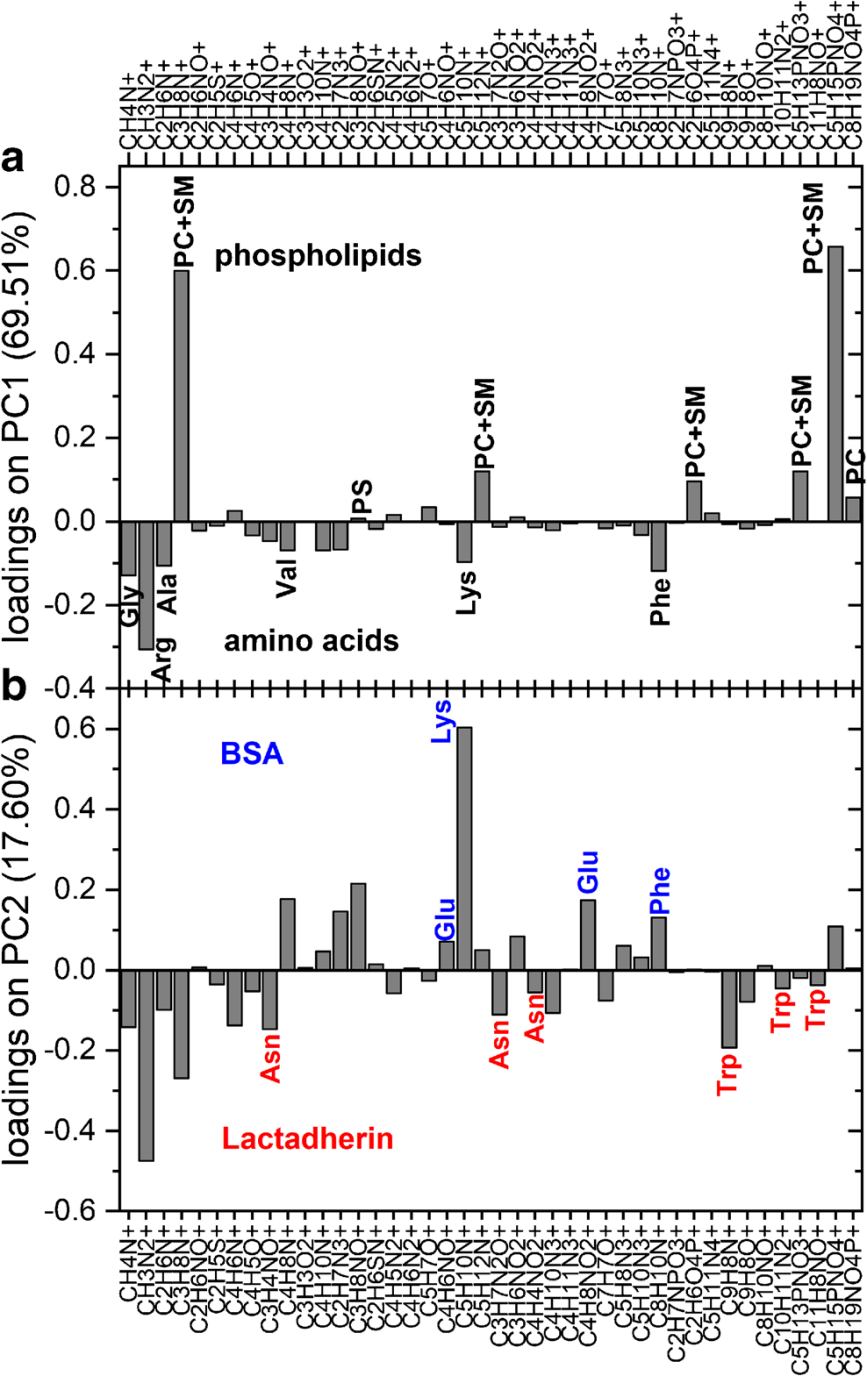
Principal component analysis (PCA) loading plots for (**a**) PC1 and (**b**) PC2. PC1 is loaded positively by ion fragments derived from phospholipids and loaded negatively by amino acid ion fragments. In turn, for PC2 ion fragments of amino acids abundant in BSA protein load in the positive direction, while fragments of amino acids with higher content in lactadherin protein load in the negative direction

In order to enhance the detection of subtle differences in the composition of multimolecular layers, formed on the silicon surface as a result of surface functionalization and binding of PMVs and their components, PCA was applied. Multivariate analysis was performed for 42 positive ion fragments originating only from biomolecules (listed in Fig. 7). The intensities of these ToF-SIMS signals that are unique to amino acids and phospholipids were examined for 60 spectra and were recorded for 9 samples: surfaces functionalized with LACT on bare GA-modified surface and on αvβ3 integrin–modified surface (this modification was analyzed separately), the same surfaces following blocking with BSA and PMV binding, as well as control samples blocked with BSA prior to and after PMV binding (Fig. 6). As revealed from the loading plot (Fig. 7a), the first principal component, which captures the majority of the variance in the data (69.51%), distinguishes between protein amino acids (negative loadings) and phospholipids (positive loadings). Therefore, the position of the data points corresponding to the PMV capturing step on the PC2 vs. PC1 scores plot, which are shifted in the direction of the positive PC1 values, clearly confirms effective PMV binding to both LACT-functionalized surfaces (Fig. 6). Additionally, the position of the control sample covered with BSA after incubation with PMVs excludes the hypothesis of non-specific PMV binding to the protein layer. In turn, further principal components capturing the residual variance uncorrelated to that described by each other and PC1 differentiate the protein composition of the examined layers. The composition of individual proteins in multimolecular layers can be described using the corresponding PC directions identified by comparison of the amino acid composition of BSA, LACT, and integrin αvβ3 proteins (see ESM Table S1). As shown in Fig. 7b, the second principal component is loaded positively with the ion fragments of amino acids that have higher abundance in BSA than in LACT or αvβ3 integrin such as lysine, glutamic acid, and phenylalanine. On the contrary, this PC is loaded negatively with the ion fragments of asparagine and tryptophan which are characteristic for LACT. Thus, PC2 separates on the PC2 vs. PC1 scores plot (Fig. 6) of the data for control samples covered with BSA from those for the LACT-immobilized sample. In turn, the data for samples with immobilized LACT were shifted after the blocking step in the direction of positive PC2 values. In addition, PC3 distinguishes the series of samples with immobilized αvβ3 integrin from the LACT immobilized on the bare GA-modified surface and the BSA control samples series (see ESM Fig. S2a). The PC3 is loaded negatively with amino acids identified as characteristic for BSA and LACT (phenylalanine, glutamic acid, tryptophan, and asparagine), whereas amino acids such as arginine, valine, leucine, and isoleucine load this principal component positively (see ESM Fig. S2a). The presented results demonstrate the unique ability of the ToF-SIMS technique supported with PCA to examine the surface molecular composition even in complex layers of several different proteins [33, 35] and PMVs.

### Orientation of surface-immobilized LACT

**Fig. 8:**
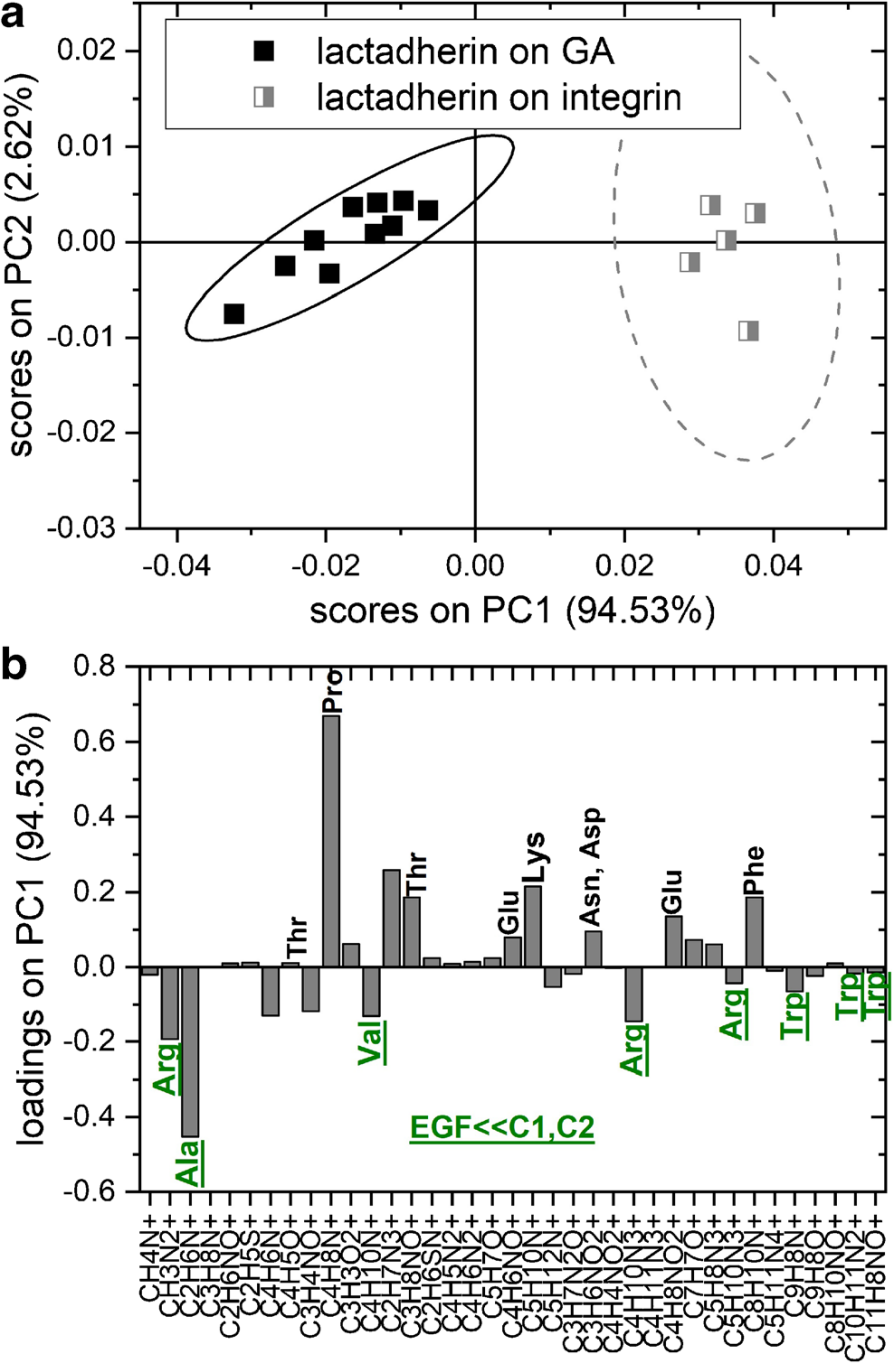
Principal component analysis (PCA) and time-of-flight secondary ion mass spectrometry (ToF-SIMS) analysis of the orientation of surface-immobilized lactadherin (LACT). (**a**) PC1 vs. PC2 scores plot of ToF-SIMS data recorded from layers of LACT functionalization on bare GA-modified surface (black squares) and on αvβ3 integrin–functionalized surface (gray squares) separates both lactadherin layers. (**b**) The loading plot for the first PC: PC1 is loaded negatively by the signals from amino acids poor in the EGF-like domain (green underlined, see ESM Table S2) and loaded positively by those abundant in EGF-like or in all domains suggesting a difference in lactadherin molecule orientation

The ToF-SIMS technique combined with multivariate analysis has already been successfully employed to determine the orientation of proteins immobilized on the surface [32]. Analysis of protein orientation is possible based on differences in the amino acid composition of particular protein domains. ToF-SIMS, with sensitivity enhanced for the outermost region of the protein layer, probes surface amino acid concentration, enabling examination of protein orientation in a more direct manner than other surface analysis methods [36, 37]. ToF-SIMS analysis of dominant orientation of surface-immobilized proteins is well-established for IgG antibody [38–42] and demonstrated also for other proteins [43–46]. Therefore, we performed PCA of ToF-SIMS data to provide an insight into the orientation of LACT immobilized following two approaches, namely, covalent binding to a GA-modified surface and site-directed binding to an αvβ3 integrin–functionalized surface. In this analysis, 37 positive ion fragments derived only from amino acids (listed in Fig. 8b) were included and PCA was developed only on the ToF-SIMS spectra recorded from LACT-functionalized surfaces before the blocking step and PMV capturing. Moreover, only an increase in the intensity of particular ions was considered as a result of the LACT immobilization step. The results of PCA involving PC2 vs. PC1 scores plot and the loadings plot for the first principal component are presented in Fig. 8a and b, respectively. As shown in Fig. 8a, the data points corresponding to different LACT immobilization strategies are clearly distinguished by the PC1 component. Analysis of the loadings plot indicates that the first PC, capturing 94.53% of variance in the data set, is associated with differences in LACT orientation. This principal component is loaded negatively with ions derived from amino acids with a low content in the EGF-like domain compared with the C1 and C2 domains such as tryptophan, arginine, alanine, and valine. In turn, PC1 is loaded positively with amino acids abundant in the EGF-like domain (glutamic acid) or in all LACT domains (lysine, proline, threonine, phenylalanine, asparagine, and aspartic acid). The amino acid composition of particular LACT domains is summarized in ESM Table S2.

## Discussion

The great potential of extracellular vesicles (EVs) has been recognized recently in both regenerative medicine and diagnostic medicine. In particular, exosomes (Ex) which belong to the population of smallest EVs (40–100 nm in diameter), derived from endosomes, have great potential in advanced drug delivery and disease treatment, and may be the next-generation delivery vehicles in near future [47–49]. MVs, as a heterogenous subpopulation of EVs, also serve to monitor platelet activity and endothelial function assessment in diagnostics [4, 6]. TEM imaging allowed to confirm the integrity of PMV membrane (the presence of lipid bilayer) after the isolation procedure. TEM and NTA size distributions showed the heterogenous population of PMVs ranging in size from 50 to 300 nm (Fig. 2c, d).

In our study, we proposed a novel approach of using the PS-binding glycoprotein-lactadherin (MFG-E8) for binding PMVs in a stereospecific and Ca^2+^-independent manner [18]. LACT immobilization was based on its properties resulting from its multidomain structure. Two different LACT-functionalized surfaces were analyzed: LACT covalently bonded to a GA surface and site-specific bonding to αvβ3 integrin, which was previously immobilized on a GA-modified surface.

The efficacy of PMV binding to LACT was monitored using the SE method, demonstrating the plausible application of optical biosensors with label-free transducers to detect traces of PMVs for both surface functionalization schemes [50]. Additionally, the functionalization steps were monitored by AFM surface characterization (Fig. 3).

Our results showed effective LACT immobilization on both surfaces and the binding of PMVs to functionalized surfaces (Fig. 4). Higher PMVs to LACT binding efficiency were observed for LACT bonded to the αvβ3 integrin–functionalized surface compared with those for LACT directly bonded to the GA-modified surface, possibly due to a lower steric hindrance and different LACT orientations. ToF-SIMS analysis of the molecular composition of the biomolecular layer after subsequent functionalization steps enabled examination of the PMV lipid composition, which revealed the presence of PS (a phospholipid characteristic for the outer surface of MVs) and a very high abundance of PC and SM lipids. This confirms the possible application of the proposed approach to analyze changes in the molecular composition of PMVs. Moreover, ToF-SIMS supported with PCA allows examination of the orientation of surface-immobilized LACT molecules.

ToF-SIMS, providing information about amino acid composition of the outermost region of protein layer (dependent on dominant proteins’ orientation), enables an examination of protein orientation in a more direct manner than other techniques determining the effective thickness of protein layer (QCM, SPR, ellipsometry, reflectometry) or single proteins’ height (AFM) [36, 37].

The discussed PCA results suggest a LACT orientation with a hidden EGF-like domain in case of LACT immobilized directly on the GA-modified surface and with all domains exposed for LACT bonded to αvβ3 integrin. One of the factors affecting LACT orientation is the surface density of the molecules; a high surface density (observed for LACT bonded to GA) promotes vertical protein orientation, whereas a low density (observed for LACT bonded to αvβ3 integrin) promotes a flat-on orientation with the maximal surface area of individual molecules [33, 51, 52]. Therefore, considering that LACT binding to αvβ3 integrin occurs via the EGF-like domain, vertical orientation with the hidden EGF-like domain and the laying orientation with all domains exposed are proposed as the dominant orientations for LACT immobilized on GA and αvβ3 integrin–functionalized surfaces, respectively. The domination of the EGF on orientation of vertical lactadherin (i.e., with the hidden EGF-like domain) on the GA-modified surface can be due to its higher reactivity at a neutral pH with the N-terminal amine groups in the EGF-like domain (deprotonated, pKa < pH) rather than with the amine groups of lysine (protonated, pKa > pH) [53] that are abundant in all lactadherin domains, and are the main substrates for the reaction with GA [54]. Moreover, the orientation with hidden EGF-like domain for LACT immobilized on the GA-modified surface was confirmed by the results of the experiment on αvβ3 integrin binding to LACT-functionalized surfaces. For LACT immobilized by its EGF-like domain through affinity binding to αvβ3 integrin–functionalized surface, no αvβ3 integrin binding was observed as expected. However, for LACT immobilized through a chemical reaction between the GA and amine group of the N-terminal located in the same EGF-like domain, the amount of αvβ3 integrin bonded was negligible but not zero (0.5(± 0.3) mg/m^2^), suggesting a LACT orientation with hindered access to the EGF-like domain.

The efficiency of microvesicles (MVs) capturing on the functionalized surface is a function of many variables and interaction between MV receptors and their ligands is very complex. Without experimental confirmation, it is difficult to predict the final effect. One of the factors is the number and availability of uptake receptors on the MV surface that is crucial for binding efficiency. To optimize the effectiveness of biofunctionalized surface, it is important to minimize non-specific binding on the exposed sensor surface and maximize the biological activity of binding sites on the surface by controlling the density, conformation, and orientation of molecules.

The proposed PMV binding surface can be readily integrated into the microfluidic system. Additionally, the proposed binding surface assimilated with a liquid biopsy-on-a-chip system for the capture and detection of PMVs and analyzing their content. However, this surface can also be characterized independently using spectral methods, such as Raman spectroscopy, infrared spectroscopy, (ToF-SIMS), or SE. Currently, the antibody-coated magnetic beads for the capture of EVs are used [55]. Nevertheless, the process of removing EVs from the surface of magnetic beads may be complicated [56]; for instance, the EV yield is often low, and during this process, EVs may lose their functionality [57, 58]. Despite the fact that centrifugation method does not allow to discard exosomes or even lipoproteins (see Fig. 2c, d), the functionalized surface allowed the capture of PMVs which are mostly enriched with PS in comparison with other EV subpopulations.

Given that the binding of MVs to the LACT-immobilized surface occurs through PS, our platform is universal compared with the surface described in our previous study [14]. Moreover, our platform is capable of capturing MVs originating from various body fluids or cell culture media. The proposed PMV capturing system can be used as a foundation to design novel POC diagnostic devices for monitoring and characterizing PMVs in clinical samples.

## Conclusion

In summary, a new LACT-functionalized surface was prepared and examined for the monitoring of PMVs. Two different strategies of LACT immobilization on a silicon surface were applied to compare different LACT orientations. A higher PMV to LACT binding efficiency was observed for LACT bonded to an αvβ3 integrin–functionalized surface compared with that for LACT directly bonded to a GA-modified surface. The proposed PMV capturing system can be used as a foundation to design novel point-of-care (POC) diagnostic devices for monitoring and characterizing PMVs in clinical samples.

## Electronic supplementary material

ESM 1 (PDF 339 kb)

## Abbreviations

AFM: Atomic force microscopy
APTES: 3-Aminopropyltriethoxysilane
BSA: Bovine serum albumin
C1: C2 and EGF (epidermal growth factor-like) domains of lactadherin
Ex: Exosomes
GA: Glutaraldehyde
LACT: Lactadherin
LOC: Lab-on-a-chip
MFG-E8: Milk Fat Globule-EGF Factor 8
MVs: Microvesicles
NTA: Nanoparticle tracking analysis
PBS: Phosphate-buffered saline
PC: Phosphocholine
PCA: Principal component analysis
PE: Phosphatidylethanolamine
PMVs: Plasma microvesicles
POC: Point-of-care
PRP: Platelet-rich plasma
PS: Phosphatidylserine
RT: Room temperature
SE: Spectral ellipsometry
SM: Sphingomyelin
TEM: Transmission electron microscopy
ToF-SIMS: Time-of-flight secondary ion mass spectrometry
αvβ3: A type of integrin
