# Supplementary material for: Using a lactadherin-immobilized silicon surface for capturing and monitoring plasma microvesicles as a foundation for diagnostic device development
Source: Anal Bioanal Chem. 2020 Sep 22;412(29):8093–106. doi: 10.1007/s00216-020-02938-5 (PMC7584542; doi:10.1007/s00216-020-02938-5)
Supplement: Supplementary file 1 — (PDF 339 kb) [file 216_2020_2938_MOESM1_ESM.pdf]

Analytical and Bioanalytical Chemistry

Electronic Supplementary Material

Using a lactadherin-immobilized silicon surface for capturing and monitoring plasma microvesicles as a foundation for diagnostic device development

Agnieszka Kamińska, Katarzyna Gajos, Olga Woźnicka, Anna Dłubacz, Magdalena E. Marzec, Andrzej Budkowski, Ewa Ł. Stępień

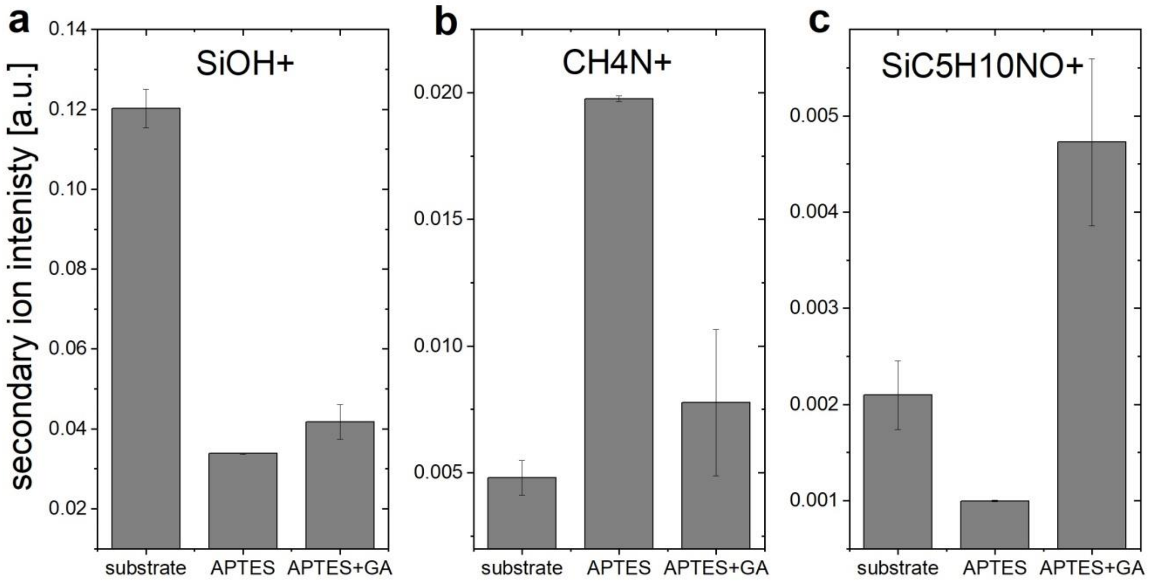

Fig. S1 TOF-SIMS characterization of silicon substrate functionalization with amino-silane (APTES) and glutaraldehyde (GA). The average normalized intensity of secondary ions derived from SiO_2 ((a) SiOH+), APTES molecule ((b) CH4N+) and APTES after reaction with glutaraldehyde ((c) SiC5H10NO+) are presented for bare substrate, silanized substrate and APTES layer after glutaraldehyde modification confirming the effective functionalization

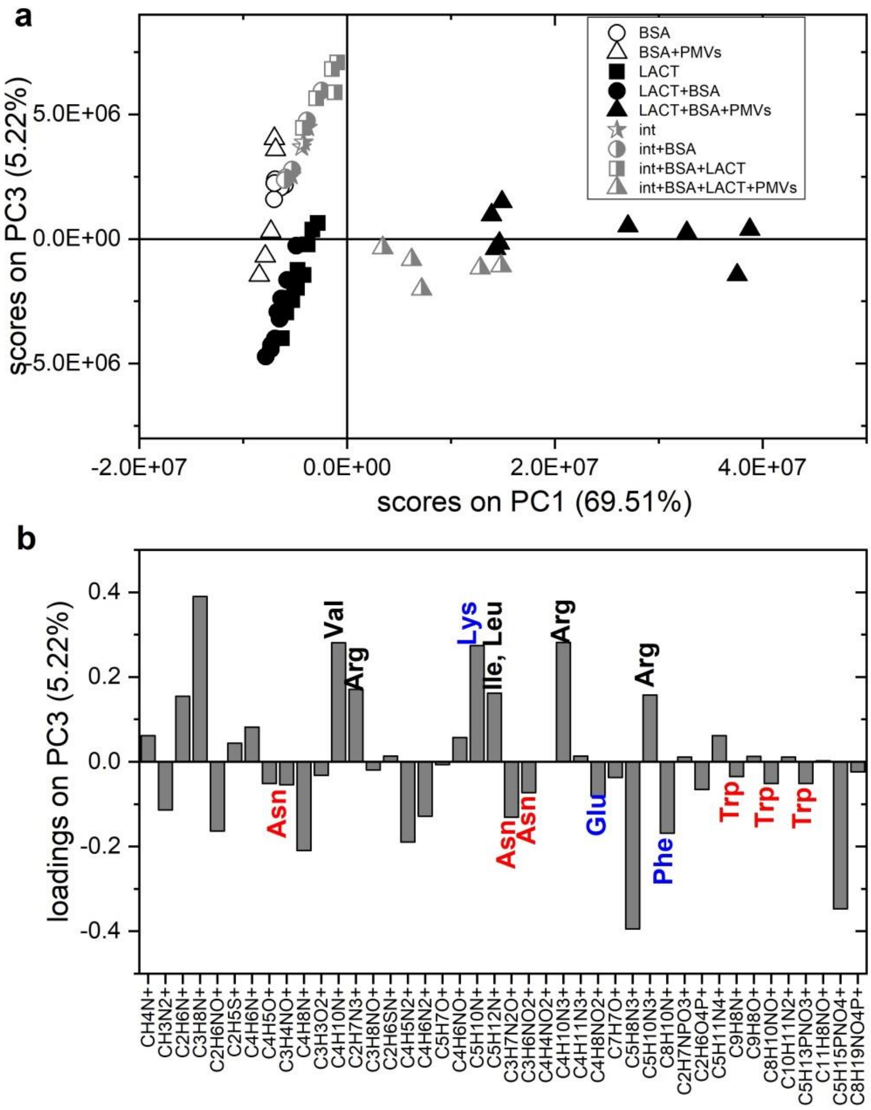

Fig. S2 Multivariate time-of-flight secondary ion mass spectrometry (TOF-SIMS) analysis of the biomolecular layer composition. (a) PC3 vs. PC1 scores plot of TOF-SIMS spectra recorded from the biomolecular layers formed on the silicon substrate after lactadherin (LACT) functionalization on bare GA-modified (black symbols) and αvβ3 integrin-functionalized surfaces (light gray symbols) following the steps of blocking with bovine serum albumin (BSA) and the binding of plasma microvesicles (PMVs). Additionally, reference samples blocked with BSA were examined (white symbols). PC1 separates protein layers from the abundant layers of PMVs, while PC3 distinguishes protein composition rich in αvβ3 integrin and LACT. (b) Loading plot on PC3

Table S1 Comparison of amino acid composition of BSA [Universal Protein Resource. http://uniprot.org. ID: P02769]. human lactadherin [Universal Protein Resource. http://uniprot.org. ID: **Q08431-1**] and αVβ3 integrin [Universal Protein Resource. http://uniprot.org. ID: P06756. P05106]

	BSA (%)	lactadherin (%)	integrin (%)
Ala (A)	7.9	8	6.2
Arg (R)	4.3	4.7	4.9
Asn (N)	2.3	8.3	4.6
Asp (D)	6.6	3.4	6.8
Cys (C)	5.8	3.6	4.1
Gln (Q)	3.3	3.4	4.5
Glu (E)	9.7	4.7	5.7
Gly (G)	2.8	7.2	7.7
His (H)	2.8	3.4	1.4
Ile (I)	2.5	3.6	4.9
Leu (L)	10.7	10.6	9
Lys (K)	9.9	4.7	5.2
Met (M)	0.8	0.8	2
Phe (F)	4.9	3.6	4
Pro (P)	4.6	4.7	5.2
Ser (S)	5.3	7.5	7.4
Thr (T)	5.6	4.7	5.2
Trp (W)	0.5	3.1	1
Tyr (Y)	3.5	3.4	3.4
Val (V)	6.3	7	6.8

Table S2 Comparison of amino acid composition of human lactadherin domains': EGF domain. C2 domain and C1 domain [Universal Protein Resource. http://uniprot.org. ID: **Q08431-1**]

	EGF	C2	C1
Ala (A)	2.3	8.2	7.5
Arg (R)	2.3	4.4	5
Asn (N)	6.8	8.8	9.3
Asp (D)	4.5	4.4	2.5
Cys (C)	13.6	1.3	2.5
Gln (Q)	2.3	4.4	3.1
Glu (E)	9.1	2.5	6.2
Gly (G)	11.4	7.5	6.2
His (H)	4.5	2.5	4.3
Ile (I)	4.5	5	2.5
Leu (L)	6.8	8.8	9.9
Lys (K)	4.5	5.7	4.3
Met (M)	0	0	1.9
Phe (F)	2.3	3.8	4.3
Pro (P)	4.5	4.4	3.7
Ser (S)	6.8	10.1	5.6
Thr (T)	4.5	4.4	5.6
Trp (W)	0	4.4	3.1
Tyr (Y)	4.5	4.4	2.5
Val (V)	4.5	5	9.9
